# Decision Making and Behavioral Choice during Predator Avoidance

**DOI:** 10.3389/fnins.2012.00125

**Published:** 2012-08-28

**Authors:** Jens Herberholz, Gregory D. Marquart

**Affiliations:** ^1^Department of Psychology, University of MarylandCollege Park, MD, USA; ^2^Neuroscience and Cognitive Science Program, University of MarylandCollege Park, MD, USA

**Keywords:** predation, escape, decision making, behavioral choice, neural circuits

## Abstract

One of the most important decisions animals have to make is how to respond to an attack from a potential predator. The response must be prompt and appropriate to ensure survival. Invertebrates have been important models in studying the underlying neurobiology of the escape response due to their accessible nervous systems and easily quantifiable behavioral output. Moreover, invertebrates provide opportunities for investigating these processes at a level of analysis not available in most other organisms. Recently, there has been a renewed focus in understanding how value-based calculations are made on the level of the nervous system, i.e., when decisions are made under conflicting circumstances, and the most desirable choice must be selected by weighing the costs and benefits for each behavioral choice. This article reviews samples from the current literature on anti-predator decision making in invertebrates, from single neurons to complex behaviors. Recent progress in understanding the mechanisms underlying value-based behavioral decisions is also discussed.

## Introduction

Successful avoidance of a predatory attack is essential for survival and future reproductive success. Failure to detect a predator before an attack initiation, failure to fight off an attack, or failure to respond to an attack with an immediate escape, can be deadly. Many aspects of nervous system function must be optimized to control anti-predator behavior, including careful sensory assessment of threat stimuli, which sometimes involves multimodal integration, rapid transmission of this information within neural structures, and finally, fast and accurate motor activation. Importantly, predator avoidance is often produced under conflicting circumstances. Many daily activities that are essential for survival, such as feeding, mate search, or habitat selection, can increase visibility and thus vulnerability to predation. Animals trying to satisfy important needs while avoiding predation face a trade-off, e.g., between eating and the risk of being eaten. Thus, the selection of the most desirable behavior requires careful calculation of costs and benefits associated with different behavioral options. For example, foraging animals must accurately measure predation risk and weigh this risk against current nutritional state. Such cost-benefit analyses are made by the nervous system through the integration of external sensory signals with current internal states, and these decisions ideally lead to behavioral choices that optimize an animal’s fitness.

Invertebrates are superbly suited to measure both the behavior and neural mechanisms underlying predator avoidance. In many invertebrates, an accessible nervous system with described neural escape circuits controls discrete escape behaviors. Thus, the link between neural machinery and behavioral expression is often identifiable and quantifiable. More recently, economic decision making, i.e., costs-benefit calculations under predatory risk, has been measured and described in a number of invertebrate species. This has opened up exciting new avenues for gaining a better understanding of complex “neuroeconomic” processes at a level of analysis not feasible in vertebrates.

The first section of this review summarizes some of the foremost examples of anti-predator behavior and underlying neural circuitry found in four different arthropods. Both the specializations and shared features of these nervous systems that allow these animals to escape immediate predatory threats are discussed. The second part focuses on economic decisions made by invertebrates in situations where the risk of predation must be carefully weighed against other vitally important needs. Finally, we suggest some important future directions for the further identification of neural mechanisms underlying behavioral decisions.

## Mechanisms of Predator Avoidance

While predators can provide direct cues such as visual or mechanosensory signals that alert prey to the presence of a predator, indirect cues, such as odors, also allow the assessment of a potential predatory threat. However, indirect cues are frequently more ambiguous and seldom provide information on the degree or immediacy of the danger posed. And indirect cues that signal the presence of a predator (although no predator is currently present) can divert attention from other vital activities or suppress these activities altogether. Different risk assessment behaviors, apprehension, and vigilance, are responses to indirect predator cues commonly described in vertebrate animals (Kavaliers and Choleris, 2001). Although they are likely to exist in invertebrates, these “anticipatory” predator avoidance behaviors are much less studied in invertebrates where the evolution of extremely fast and powerful escape reactions in response to immediate attack has arguably reduced the necessity for extensive predator scanning and risk assessment. Additionally, while numerous behaviors in an animal’s repertoire contribute to predator avoidance, most are subtle and difficult to subject to neurobiological analysis. For instance, an animal’s decision when and where to forage is greatly shaped by the risk of predation (Lima and Dill, 1990). How an animal calculates this predatory risk and weighs it against concurrent internal and external demands is certainly an interesting question; however, the time-scale and context of such a decision make it difficult to subject to detailed electrophysiological or neuroanatomical analysis. Instead, what has overwhelmingly sufficed for the study of predator avoidance in neuroscience has been the analysis of much more discrete escape or startle behaviors. Because escape behaviors are so critical, they must interface with and frequently override the performance of any ongoing or planned behaviors. And while other behaviors may have a greater evolutionary importance over the long term, seldom are they as time-sensitive and unforgiving as escape. Thus, it is unsurprising that the circuits tasked with the sensory acquisition, computation, and action upon salient predatory cues are frequently the largest, most robust, and most highly stereotyped neural systems in an organism.

If a predator is around, it is critical to identify and react to predatory cues at an appropriate time and in an effective manner. Consequently, escape behaviors must be fast, accurate, and robust in order to be effective countermeasures against the often rapid predatory behaviors they combat. It is believed that the time-sensitive nature of these behaviors necessitates a small number of large elements in order to both maximize conduction velocity and minimize synaptic delay. Thus, escape circuits commonly have “giant fibers (GFs),” frequently the largest axons in an animal’s nerve cord, which can be readily identified by their size, location, or morphology. These characteristics allow for rapid identification and often make these neurons accessible to a wide range of cell biological and electrophysiological studies.

Because of their simplicity and clear function, these circuits have been excellent models for the study of the neural basis of behavior. Recent work, however, has uncovered a surprising degree of flexibility not previously recognized in these “simple,” “reflexive” systems. High-speed video recordings have exposed a previously unappreciated level of complexity to arthropod escape behaviors that has made researchers question the structure and even identity of the underlying circuits that were originally assumed to be responsible for escape (Hammond and O’Shea, 2007a,b; Card and Dickinson, 2008a,b; Fotowat et al., 2009). Additionally, wireless-recording techniques have been adapted to small invertebrate models allowing, for the first time, the correlation of neural activity from multiple identified neurons with the time-course of escape behavior in unrestrained preparations (Fotowat et al., 2011; Harrison et al., 2011). And while neural-behavioral correlations are not uncommon, escape behavior in invertebrates provides possibly one of the few opportunities to simultaneously record from all the critical elements in a neural circuit and relate it to what is now appreciated as an increasingly complex, but still tractable, behavior. This provides quite possibly one of the best current opportunities for the comprehensive analysis of the neural underpinnings of decision making surrounding a behavior.

While there is likely a broad spectrum of complexity in the circuits embedded in even the most simple nervous system, escape circuits in invertebrates are frequently divided into two broad categories: those that contain “command” or “command-like” elements and those that do not (Kupfermann and Weiss, 1978, 2001; Edwards et al., 1999; Eaton et al., 2001). In command systems, the activity of the command neuron is thought to be necessary and sufficient for the production of a behavior. Often a single spike in this neuron is sufficient for the readout of an entire escape program. While highly adaptive, these rapid behaviors are highly stereotyped, showing little variability. In contrast, the escape behaviors produced by systems ostensibly lacking a command element typically display a greater degree of complexity and flexibility and are frequently made up of a sequence of independently variable components. This flexibility affords the animal a greater degree of control over the precise timing, direction, and structure of the escape behavior. Traditionally, however, this is assumed to come at an additional computational cost that adds to the latency of the action (Bullock, 1984). Alternatively, variability may be added to behavioral decisions by sequential neural processing. For example, in the medicinal leech decision neurons can be active during competing behaviors (e.g., swimming and body shortening), and stimulation of one decision neuron can produce two different behavioral outputs, swimming and crawling. Hypothesized to be organized in a hierarchical order, the first neuron in the chain would drive general behavioral action, the next one would command selection from a pool of discrete motor patterns, and the next one would initiate the most desirable behavioral choice (Esch and Kristan, 2002).

### Giant-neuron mediated escape

#### Crayfish

Crayfish are equipped with powerful escape reactions mediated by rapidly responding neural circuits (reviewed in Wine and Krasne, 1982; Krasne and Wine, 1984; Edwards et al., 1999). These circuits control at least three distinct motor programs that propel the animals in different directions, but always away from real or assumed threats. Circuits and their associated tail-flips can be divided into two major categories, giant and non-giant. Two circuits, the lateral giant (LG) and medial giant (MG) system contain giant interneurons as key “command” components, are made for speed, and require strong and phasic input for their activation. In contrast, a poorly elucidated non-giant system is believed to control slower, but more variable escape tail-flips (Edwards et al., 1999). These escape circuits have been the focus of 65 years of intensive research since they were first described by Wiersma (1947, 1952) in his pioneering work.

The LG interneurons, two large fibers consisting of a series of gap junction-linked neurons that project from tail to head, are activated by tactile and strong hydrodynamic stimulation of sensory hairs and proprioceptors located on the abdomen. The LG interneurons also receive excitatory inputs from rostral sensory organs, but these inputs alone are insufficient to fire the LG. If these inputs sum with strong caudal inputs, however, a single LG action potential (in one of the two fibers) is sufficient to produce an escape motion that thrusts the animal upward and away from the point of caudal stimulation (Liu and Herberholz, 2010). The motor program is activated within milliseconds after stimulation and speed and accuracy is guaranteed through several structural and functional specializations within the circuit (Herberholz et al., 2002). Once activated, the LG interneurons drive giant motor neurons via rectifying electrical synapses, which activate fast flexor muscles in the last two thoracic and first three abdominal segments causing a bending of the abdomen around the thoracic-abdominal joint and thus the stereotyped “jack-knife” motion that propels the animal upward (Wine and Krasne, 1972). Latency is minimal, with 5–15 ms between stimulation and start of the behavioral response, and varies according to both internal (e.g., animal size: Edwards et al., 1994) and external conditions (e.g., water temperature: Heitler and Edwards, 1998). This short latency is accomplished by the high transmission velocity due to the diameter of the GFs and by electrical coupling among most circuit components (Figure 5A).

The MG interneurons, a pair of large fibers projecting from head to tail, are activated by strong, phasic visual or tactile inputs directed to the front of the animal. The MG interneurons receive their excitatory inputs in the brain where both neurons are electrically coupled to each other. One action potential in one of the MGs is sufficient to drive the fast and stereotyped backward escape response. The MG interneurons connect electrically to giant motor neurons, which activate fast flexor muscles in all abdominal segments, causing the bending of the entire abdomen and propelling the animal backward away from the point of stimulation. MG tail-flips in response to tactile stimulation are as fast as LG-mediated tail-flips and happen within a few milliseconds (Wine and Krasne, 1972). Visually activated MG tail-flips are slower, but are still produced as quickly as 50 ms after detection of a visual danger stimulus (Liden and Herberholz, 2008; Liden et al., 2010).

Non-giant-mediated tail-flips are controlled by a circuit that lacks giant interneurons. These tail-flips are elicited by a variety of different stimuli, typically more gradual and less forceful in presentation than those activating giant-mediated tail-flips. They are produced with longer latencies, usually up to 10-fold slower than giant-mediated tail-flips, and considered, in a way, “voluntary” because the animal “chooses” to activate certain patterns of fast flexor muscle groups. Thus, the timing and direction of non-giant tail-flips can be modulated, resulting in a much more variable behavior compared to the giant-mediated tail-flips (Wine and Krasne, 1982; Wine, 1984). Non-giant tail-flips are also used during “swimming,” where a series of tail flexions and extensions propels the animal backward through the water.

**Figure 1:**
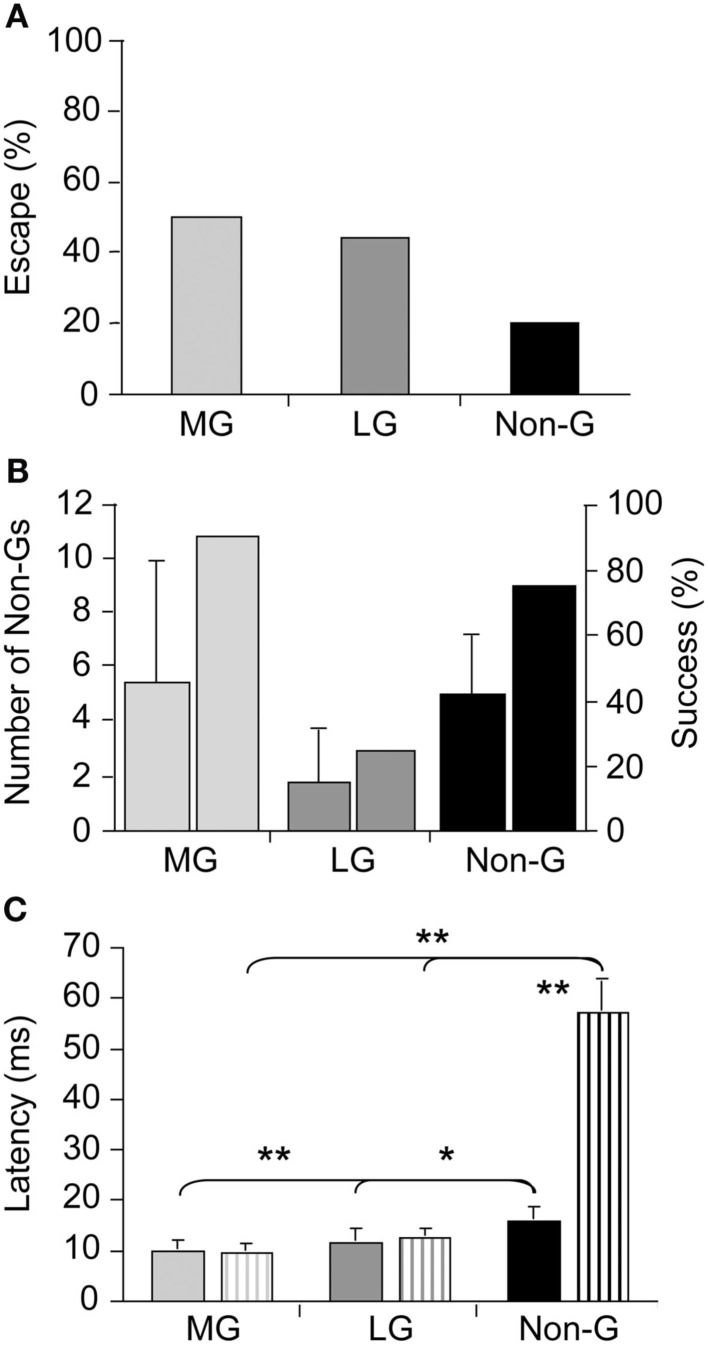
**Escape success and latencies measured in juvenile crayfish attacked by dragonfly nymphs**. **(A)** Attacks evoking tail-flips mediated by the medial giant (MG) or lateral giant (LG) interneurons are equally effective to prevent capture whereas attacks eliciting non-giant (Non-G) tail-flips are much less effective. **(B)** Unsuccessful MG and Non-G, but not LG responses are frequently followed by a series of Non-G tail-flips (left bars), which substantially increase the overall rate of escape (right bars). **(C)** Escape latencies for crayfish attacked by predators (solid bars) or stimulated with a handheld probe (striped bars) are similar for giant mediated (MG and LG) tail-flips, but significantly shorter for predator evoked Non-G tail-flips. Modified from Herberholz et al. (2004).

Although our understanding of the neural underpinnings of tail-flip escape, especially tail-flips produced by the LG circuit, is extensive and essentially unmatched by that of other experimental models, our knowledge of escape circuit activation in response to real predatory danger is virtually non-existent. Using dragonfly nymphs as natural predators, Herberholz et al. (2004) showed that all three escape circuits of juvenile crayfish were activated in response to attacks (Figure 1A). Initial escape responses to predatory strikes were primarily mediated by giant tail-flips; frontal attacks evoked MG tail-flips whereas attacks directed to the rear of the crayfish elicited LG tail-flips. While few attacks elicited non-giant tail-flips initially, overall escape performance improved substantially when non-giant tail-flips were produced following capture. Overall, crayfish were successful at evading dragonfly nymphs, avoiding the predator’s strike with giant tail-flips in 50% of all cases and escaping, after being captured, using a series of non-giant tail-flips in more than 75% of the remaining cases (Figure 1B). Interestingly, latencies for non-giant tail-flips that were produced as initial response to the predator strike were much shorter than latencies of non-giant tail-flips elicited by tactile stimulation with a handheld probe (Figure 1C). This suggests that crayfish prepared the non-giant escape before the strike was delivered, possibly integrating visual and hydrodynamic cues from the approaching predator in anticipation of the attack. The study also revealed that crayfish relied entirely on their fast and powerful tail-flip escape behaviors; crayfish showed no signs of predator recognition, vigilance, or avoidance behaviors in any of the trials (Herberholz et al., 2004). Thus, the decision to escape, at least from this specific predator, is based on the activation of fixed action patterns elicited by predatory stimuli. The decision to escape is made at individual decision-making neurons; if the predatory signal is sufficient to activate them, escape will inevitably follow.

#### Drosophila

There are a number of similarities between the GF system in *Drosophila* and the MG system in crayfish. Like the MG system, the GF system contains GFs originating in the brain that project down contralaterally to primary motor neurons that control the thoracic musculature responsible for the fruit fly’s escape behaviors (reviewed in Wyman et al., 1984; Allen et al., 2006). In these giant fibers, a single spike is normally sufficient for the activation of an escape jump followed by flight initiation. Despite the motor portion of both the MG and GF being well described, comparatively little is known about the visual and mechanosensory pathways that feed into the giant fiber systems of either animal (Figures 5A,B).

While the escape behaviors produced by these circuits are extremely fast due to high conductance velocities and the minimal synaptic delay from a preponderance of electrical synapses, this speed has generally been thought to come at the expense of flexibility (Bullock, 1984). Thus, giant-mediated escape behaviors are traditionally characterized as highly stereotyped with little variance in timing or direction; and whatever variance the result of stochastic properties of the system and not the consequence of neural computation (Bullock, 1984).

Although *Drosophila* has been a preeminent genetic model since the start of the twentieth century, its diminutive size limited its use in electrophysiology until the 1970s (Bellen et al., 2010). And while the GF system was identified in 1948 (Power, 1948), it was not electrophysiologically characterized and linked to the production of escape behavior until the early 1980s (Wyman et al., 1984). This escape behavior was initially characterized as an abbreviated form of “voluntary” flight initiation (Trimarchi and Schneiderman, 1995a). While voluntary flight initiation is preceded by a series of postural adjustments that prepare the fly for stable, directional flight, escape flight lacks these preflight postural leg, and wing movements. Instead, escape initiation consists almost exclusively in the extension of the fruit fly’s mesothoracic legs that propels the insect off of the substrate, which is only then followed by the unfolding and initiation of wing movements (Card and Dickinson, 2008a).

**Figure 2:**
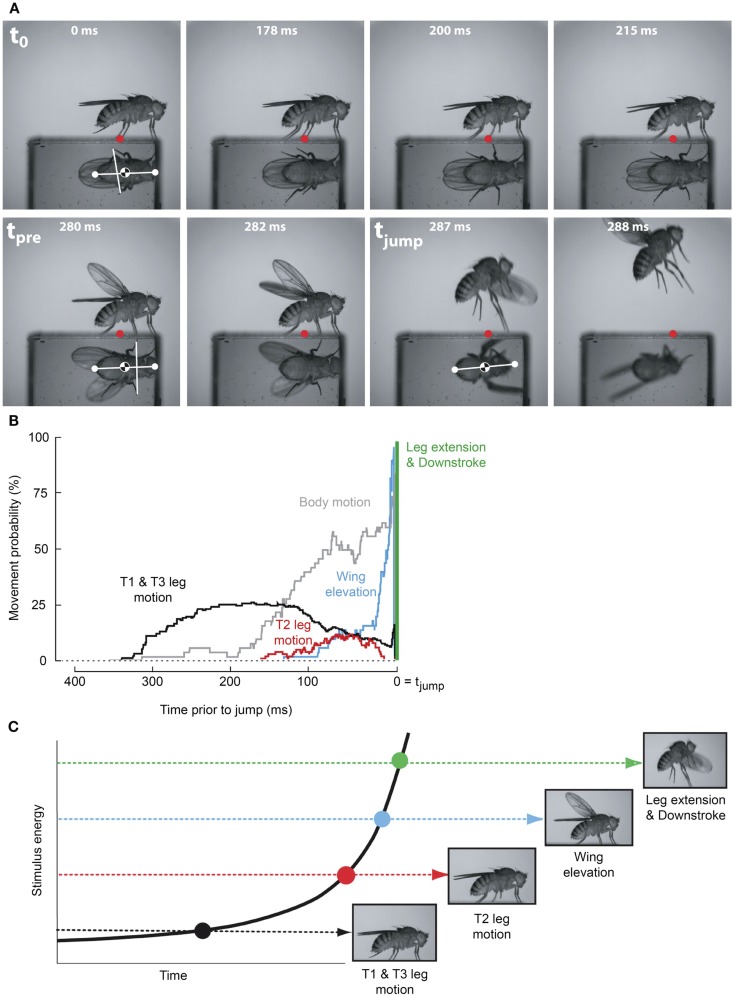
**Escape flight planning and execution in *Drosophila***. **(A)** High-speed video sequence shows a typical escape to a looming frontal stimulus with a prism allowing for simultaneous observation of ventral and side profiles. Time stamps are milliseconds elapsed since stimulus onset. Red dots mark the initial contact point of the second leg tarsi with substrate. White dots mark head and abdomen points. **(B)** Probability that body parts of the fly (black, T1 and T3 legs; red, T2 legs; blue, wings; gray, body) were moving prior to takeoff (green line). **(C)** As stimulus intensity increases, independent motor programs are activated eliciting discrete escape subbehaviors prior to takeoff. Adapted with permission from Card and Dickinson (2008b).

As the GF system was the only identified *Drosophila* escape circuit, it was assumed to mediate the escape behavior elicited by all visual, chemical, and mechanosensory stimuli that elicit an escape jump (McKenna et al., 1989). However, a number of observations have accumulated that conflicted with this canonical interpretation. For instance, in the housefly GF activity was shown not to be necessary for the production of an escape jump in response to looming stimuli (Holmqvist, 1994). Additionally, Trimarchi and Schneiderman (1995b) provided evidence for an olfactory-induced flight initiation reminiscent of the fruit flies’ escape behavior that was also not mediated by the GFs. More recently, the simplicity of the observed escape behavior was reassessed through high-speed video analysis (Hammond and O’Shea, 2007a,b; Card and Dickinson, 2008a,b). This work illustrated that these “simple” escape behaviors were far more complex and nuanced than originally assumed (Figures 2A,B). Card and Dickinson (2008a) showed that rather than a simple escape jump, the escape behavior in wild-type fruit flies involves a complex sequence of events consisting of at least four distinct subcomponents: an initial freeze followed by postural adjustments, wing-elevation, and finally an escape jump coordinated with the initial down stroke of flight initiation (Figure 2C). These behaviors do not appear to merely be a fixed action pattern as new information continues to be integrated into and affect subsequent components of the behaviors even after sequence initiation (Hammond and O’Shea, 2007b). These preflight behaviors were found to influence both the trajectory as well as initial flight stability of the escape behavior (Card and Dickinson, 2008b).

This newly appreciated complexity of the response suggests that this escape behavior is either not in fact mediated by the GF system or that additional unidentified pathways must be involved that are responsible for the preflight sequence that proceeds the escape jump (Card and Dickinson, 2008b). Toward this end, evidence for a previously unknown escape circuit was recorded by Fotowat et al. (2009). In the absence of GF activation, the activity of this novel circuit correlated with the production of escape behavior in response to looming stimuli. While this pathway is yet to be anatomically identified, its activity shares features similar to well-described circuits responsive to looming stimuli in both vertebrates and invertebrates (e.g., pigeon: Sun and Frost, 1998; locust: Rind and Simmons, 1992; crab: Oliva et al., 2007; bullfrog: Nakagawa and Hongjian, 2010). All of this strongly suggests that the GF system is not necessary for the production of escape behavior in the fruit fly, but that the GF system, possibly akin to the escape circuits in the crayfish, may be one of many present in *Drosophila*.

Being that sudden changes in luminance (light-off) are the only stimulus to reliably produce GF-mediated escape behavior, and then only in white-eyed fruit fly mutants, what role, if any, that the GF system plays in actual escape behavior of wild-type fruit flies is now unclear. Although stimuli that reliably recruit the GF system in wild-type flies are unknown, it seems unlikely that the GF system is simply the vestige of a lost escape circuit. While the newly identified looming sensitive pathway might be tuned to a selective set of stimulus features, the GF system could still serve as a robust, broadly tuned escape circuit capable of producing rapid escape behavior when more selective systems fail (Fotowat et al., 2009).

### Visual interneuron mediated escape

#### Locust

**Figure 3:**
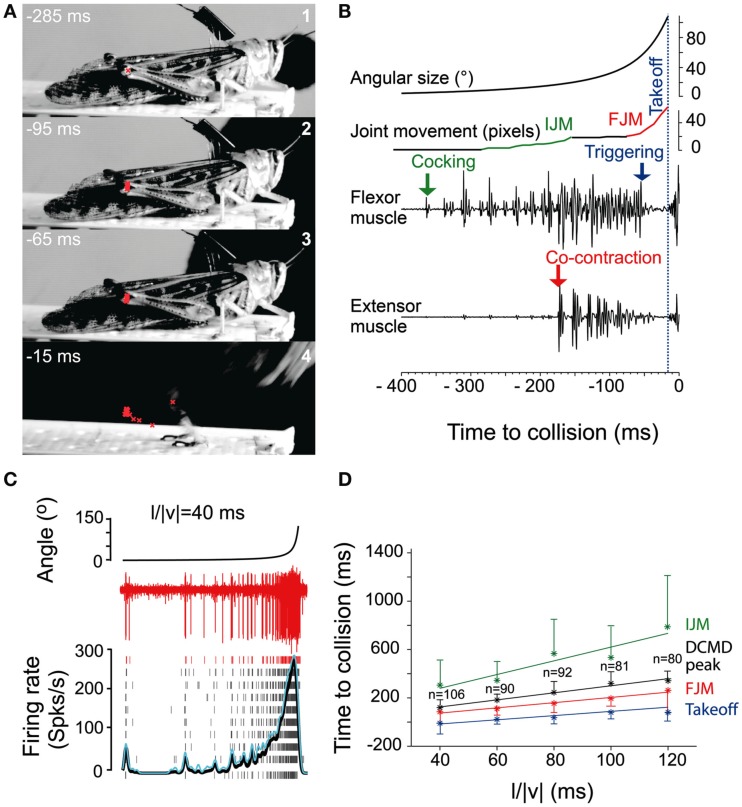
**Escape jump and DCMD activity in locusts in response to looming stimuli**. **(A)** Four high-speed video frames from a locust producing an escape jump with time to collision listed in milliseconds. The position of the femur-tibia joint is marked in red to calculate pixel movements of the joint. **(B)** Muscle recordings from the same trial. Stimulus angular size is shown on top with joint movements and flexor and extensor recordings below. (IJM, initial joint movement; FJM, final joint movement.) **(C)** DCMD activity measured extracellularly in the nerve cord from one locust (red traces). Raster plots show DCMD spikes recorded in 10 repetitions of the stimulus. Black and blue traces show average DCMD firing rate and its standard deviation, respectively. **(D)** Timing of joint movements, DCMD peak and takeoff obtained from seven locusts. The DCMD peak occurred after the IJM and before the FJM and takeoff for all *l*/|*v*| values (*l*/|*v*| = ratio of stimulus radius (*l*) to the velocity (*v*) of the stimulus). Adapted with permission from Fotowat and Gabbiani (2007).

While locusts produce avoidance behavior in response to a variety of noxious stimuli (Riede, 1993; Friedel, 1999), the best studied of these are escape jumps in response to looming stimuli (reviewed in Pearson and O’Shea, 1984; Burrows, 1996; Figure 3). Like the escape behavior of fruit flies, the locust escape jump is a complex behavior composed of a sequence of distinct components, which allow the animal to direct this jump (Santer et al., 2005b). In preparation for a jump, tilting postural movements mediated by the pro- and mesothoracic legs rotate the long axis of the locust toward the direction of the eventual jump (Hassenstein and Hustert, 1999; Santer et al., 2005b; Figure 3A). The actual jump is produced through the cocking of the hindlegs, storage of energy by the co-contraction of tibia flexor and extensor muscles, and finally the release of this energy, triggered by flexor inhibition (Burrows and Morris, 2001). Given the time required to store sufficient energy in the animal’s hindlegs, co-contraction must begin as soon as possible in order to allow for a timely escape. In contrast, the adjustment of pro- and mesothoracic limbs can continue throughout co-contraction, allowing for alterations of escape trajectory up until the escape jump is triggered (Santer et al., 2005b). On the other hand, if the hindlegs were used to control direction, it is thought that the decision of where to jump would have to be made over 100 ms before the jump is produced.

Not only are locusts able to direct these jumps up to 50° to either side of their long axis, but their escape circuitry allows them to control the timing, distance, and elevation of these jumps (Santer et al., 2005b; Simmons et al., 2010). Similar to *Drosophila*, this complex sequence of events does not appear to be a fixed action pattern that once initiated must be taken to completion as the locust can relax this co-contraction and release the stored up energy without the production of an escape jump (Heitler and Burrows, 1977).

Motor areas controlling these escape jumps are innervated by a pair of large interneurons, the descending contralateral movement detectors (DCMDs) which receive excitatory inputs from lobula giant movement detector (LGMD) neurons that are responsive to looming stimuli. With a one-to-one relationship with the LGMDs, the DCMDs produce action potentials in response to looming stimuli, with their firing rate increasing as the looming object gets closer. Thus, the DCMDs were originally thought to play a major role in jump production, sometimes compared to the giant fibers in crayfish and fruit flies that control their fast escape maneuvers (Burrows, 1996). However, locusts prepare for jumps by co-contracting flexor and extensor tibiae muscles for ~100 ms before the jump is released by relaxation of the flexor muscles. Thus, the jump is not simply triggered by suprathreshold excitation of the DCMDs, because withdrawal of excitation and inhibition are needed during the preparatory phase of the jump (Figure 3B). Nevertheless, the DCMDs seem to participate in all phases of the jump. Fotowat and Gabbiani (2007) compared electrophysiological recordings with high-speed video recordings and found that the rising phase of the firing rate of the DCMDs coincided with the preparatory phase of the jump, whereas the peak firing rate coincided with the co-activation period of flexor and extensor muscles, and decay of firing rate to less than 10% coincided with takeoff. This suggests that different stages of jump production could be controlled by distinct phases in the firing pattern of the DCMDs (Figures 3C,D). Hindleg flexion in preparation for the jump, however, is not dependent on DCMD activity. When the connective containing the DCMD neuron was severed, hindleg flexion still occurred, and it could also be evoked with visual stimuli that did not cause high firing activity in the DCMDs. This showed that while the activity of the DCMDs may contribute to hindleg flexion, it was not necessary for it and, thus, other descending pathways would seem to be involved (Santer et al., 2008). Using a telemetry system to record DCMD and motor neuron activity in freely behaving locusts, it was found that the number of recorded DCMD spikes predicted motor neuron activity and jump occurrence, and the time of peak firing rate predicted time of takeoff (Fotowat et al., 2011). Although this underlined the role of the DCMDs as neurons exhibiting discrete firing responses to looming stimuli, which in turn affected discrete stages of escape motor output, jump production remained intact, and occurred at the same time as in control animals following DCMD ablation. Thus, another neuron for jump production must exist, and this may be the descending ipsilateral movement detector neuron (DIMD), which responds to looming targets, similarly to the DCMD (Fotowat et al., 2011). Additionally, another descending interneuron that responds to looming stimuli has recently been described. Thus visually mediated escape behavior in locusts is likely controlled by at least three different descending neurons (Gray et al., 2010). How these neurons interact to produce the escape behavior remains to be determined (Figure 5C).

Locusts also produce an avoidance behavior during flight. When looming stimuli are presented, flying locusts produce a gliding dive similar to the dives used by other insects to evade aerial predators. After DCMD neurons are activated by a looming stimulus, they produce short-latency excitatory postsynaptic potentials (EPSPs) in a motor neuron that raises the wing into the gliding posture. Stimuli that evoked high-frequency firing in the DCMDs also reliably elicited the gliding response, and the behavior was less frequently observed when high-frequency DCMD spikes were absent (Santer et al., 2005a). However, similar to the escape jump, DCMD activity was not always sufficient to evoke gliding. Most likely, its high-frequency activity must be precisely timed with wingbeat phase because glides can only be produced during wing elevation. In addition, other neurons that are implicated in jump production (e.g., the DIMDs) may also be involved in escape gliding in flying locusts (Santer et al., 2006).

#### Crabs

The role of identified neurons in visually mediated escape behavior has also recently been studied in grapsid crabs (reviewed in Hemmi and Tomsic, 2012). The firing rate of these motion-sensitive lobula giant (LG) neurons in response to looming stimuli corresponds with the intensity of the crab’s escape behavior. Four distinct classes of these neurons have been anatomically and physiologically described. All four classes show wide-field tangential arborization in the lobula, somata located beneath, and axons that project toward the midbrain; however, they are uniquely identifiable due to differences in morphology and response preferences (Medan et al., 2007).

**Figure 4:**
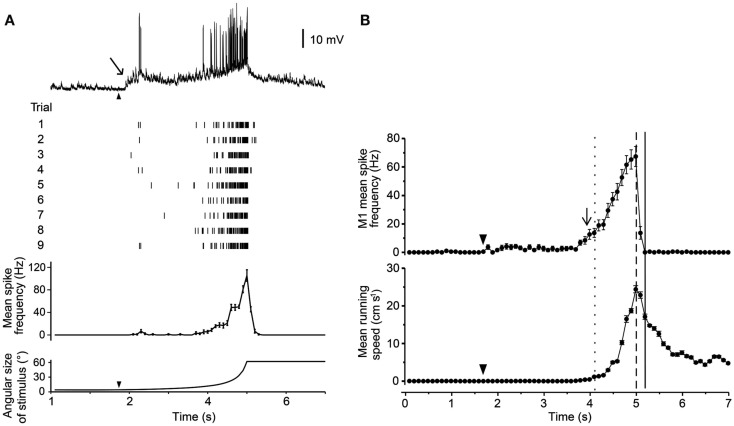
**Response of a crab’s LG neuron to looming stimuli and correlation with escape run**. **(A)** Intracellular trace from one LG neuron in response to a looming stimulus. Raster plot shows responses from one neuron to nine repetitions of the stimulus. Histogram shows mean spike rate obtained from all nine trials. Angular size of the looming object is shown in bottom trace. **(B)** Mean spike rate from a single LG neuron (top) and mean escape running speed (bottom). Arrowheads mark the start of stimulus expansion and long arrows mark increase in spike rate above resting level. Adapted with permission from Oliva et al. (2007).

Three of these LG classes receive proprioceptive inputs from the legs, and thus could potentially integrate some contextual information during predator escape (Berón de Astrada and Tomsic, 2002). Oliva et al. (2007) tested the escape behavior of grapsid crabs on a freely rotating styrofoam ball and recorded escape movements (i.e., running) while looming stimuli were presented. They also recorded intracellularly from the LG neurons in restrained crabs and compared these recordings with the behavioral data. Escape runs were initiated soon after the LG neuron increased its firing rate, and after maximum stimulus expansion, the LG neurons stopped firing, coinciding with run deceleration in freely behaving animals. Moreover, the spike frequency of the LG neurons reflected the timing and speed of the escape response (Figures 4A,B). Interestingly, the activity of the LG neurons is strongly affected by season with responses weaker in winter when predation risk is typically low and the animals are less active (Sztarker and Tomsic, 2008).

**Figure 5:**
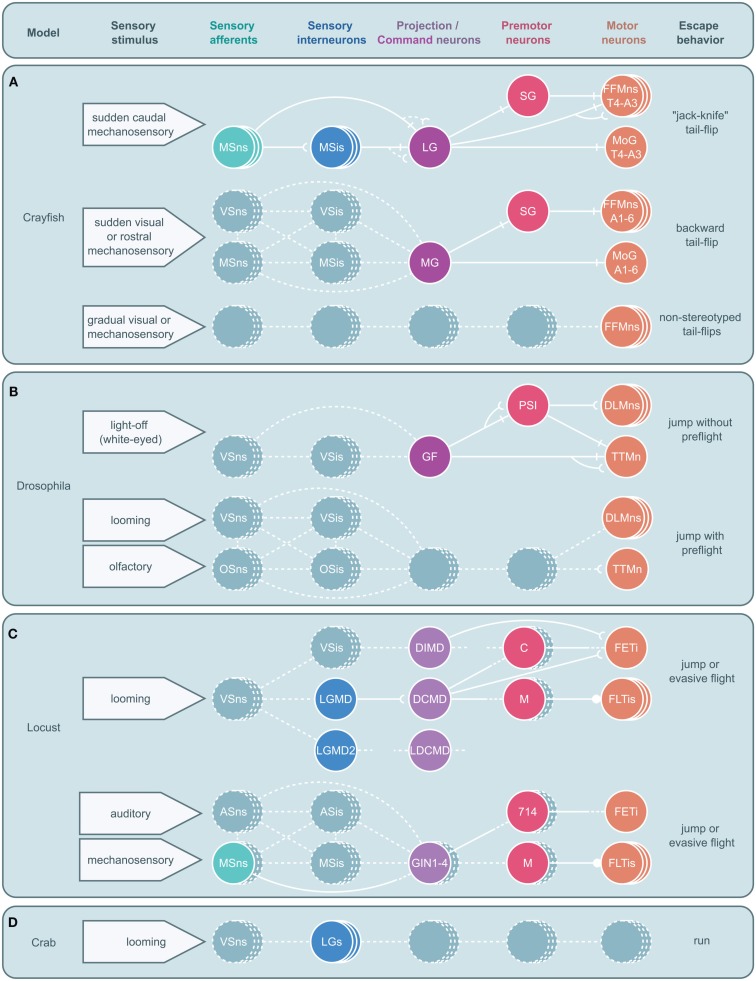
**Circuitry for arthropod escape behavior**. Neural circuits underlying escape behaviors for crayfish **(A)**, *Drosophila* **(B)**, locust **(C)**, and crab **(D)** are illustrated. Circuits are divided into five levels: sensory neurons, sensory interneurons, projection (ascending or descending) or command neurons, premotor neurons, and motor neurons with associated sensory stimuli on the left and motor output on the right. Solid circles and lines represent identified neurons and connections while dashed circles and lines represent neurons and connections yet to be identified. Stacked circles represent a population of neurons. Lines end in four ways: with a perpendicular line, a concave cup, a circle, or dashes. Perpendicular lines represent electrical synapses. Concave cups represent electrical synapses. Circles represent inhibitory synapses. Dashes indicate an unknown synapse type. Generic abbreviations: MSns, mechanosensory neurons; MSis, mechanosensory interneurons; VSns, visual sensory neurons; VSis, visual sensory interneurons; OSns, olfactory sensory neurons; OSis, olfactory sensory neurons; ASns, auditory sensory neurons; ASis, auditory sensory interneurons. **(A)** Crayfish tail-flips are controlled by one of three circuits, the lateral giant (LG), medial giant (MG), and non-giant escape circuit. While the LG system is almost fully elucidated and the abdominal motor outputs of the MG are also well described, very little beyond the fast flexor motor neurons (FFMns) are known to play a part in non-giant tail-flips. SG, segmental giant neuron, MoG, motor giant neuron. **(B)** *Drosophila* escape jumps are the result of at least two circuits; a giant fiber (GF) system mediating jumps lacking preparatory leg and wing movements and a yet to be identified escape circuit that produces escape jumps with preparatory preflight limb and wing adjustments. (PSI, peripherally synapsing interneuron, DLMns, dorsal lateral motor neurons, TTMn, tergotrochanteral muscle neuron.) **(C)** Locusts possess at least two escape circuits as well, one responsive to looming stimuli and another responsive to auditory and mechanosensory stimuli. While numerous neurons that are believed to play a role in these behaviors have been identified, both circuits remain incomplete. [LGMD, lobula giant movement detector neuron; LGMD2, lobula giant movement detector neuron 2, DCMD, descending contralateral movement detector neuron; DIMD, descending ipsilateral movement detector neuron; LDCMD, late descending contralateral movement detector neuron, C, C (“cocking”) neuron, M, M-neuron, FETi, fast extensor tibia motor neuron, FLTis, flexor tibia motor neurons, 714, neuron 714.] **(D)** In crabs, a class of visual interneurons, the lobula giants (LGs), have been identified that are thought to play a role in the crab’s escape behavior; however, no other elements in this escape circuit have been elucidated.

The relation between LG neuron activity and escape behavior was also nicely demonstrated in experiments that tested short-term and long-term visual memory in crabs. Tomsic et al. (2003) showed that LG neurons changed their responses to a visual threat (displacement of a black screen above the animal) in correspondence with the behavioral changes observed in unrestrained animals. Modification of LG neuron activity occurred during learning and persisted, after spaced training, for 24 h. However, while the memory of freely behaving crabs reflects a strong stimulus-context association, LG neurons generalize the learned stimulus into new spatial locations. Thus, despite being able to clearly distinguish the learned stimulus from other similar stimuli (i.e., stimulus memory), the LG neurons do not appear to be involved in processing contextual visual information (i.e., where the stimulus was learned; Sztarker and Tomsic, 2011). In summary, the LG neurons are sensory neurons located in the eyestalk, and their neural activity patterns closely match escape behavior produced in unrestrained crabs (Medan et al., 2007). Their exact role in producing the behavior, however, is unknown. To answer this question, detailed investigation of the descending pathways that connect the LG neurons to the motor centers that control escape runs will be required (Figure 5D).

## Value-Based Decision Making

Adaptive behavioral decisions are essential for the survival and reproductive success of most animals, including humans. Animals can typically choose from several behavioral alternatives, which need to be evaluated before the most desirable option is selected. To determine what behavior is most desirable at any given point, the nervous system must integrate external conditions (e.g., predation risk) with current internal drives (e.g., hunger state), thus trading off the costs and benefits of different alternatives before deciding which one to choose. For example, a hungry animal is more likely to choose a behavioral option that involves risks because the value placed on foraging is greater than the value placed on other alternatives such as hiding. If the benefit of finding a meal outweighs the estimated cost of being attacked by a predator, the decision is to forage. If the value placed on foraging is low because the animal is satiated, other behavioral options become more valuable and behavioral output will shift toward less risky activities. The literature on value-based decision making, especially with a focus on prey behavior in predator-prey interactions, is extensive and covers a wide range of organisms (e.g., Ydenberg and Dill, 1986; Lima and Dill, 1990).

The relatively new field of “neuroeconomics” is concerned with the neural underpinnings of value-based decision making in humans and other non-human primates (Schall, 2001; Rangel et al., 2008) and there is now fast growing interest in understanding the neural mechanisms that govern cost-benefit calculations. An increasing number of studies performed in humans and other primates are combining non-invasive techniques such as functional magnetic resonance imaging or cortical recordings with discrimination tasks or cognitive experiments (Glimcher and Rustichini, 2004; Huettel et al., 2005; Sugrue et al., 2005). The complexity of the mammalian brain, however, presents many challenges. It is difficult to directly correlate neuronal activity and behavioral expression and to obtain detailed information on neural circuit organization, cellular mechanisms, and the interplay between sensory and motor systems. Decision-making circuitry has been studied quite extensively in various invertebrates, but descriptions of neural mechanisms underlying value-based (economic) behavioral decisions are rare (Kristan and Gillette, 2007; Kristan, 2008). This is surprising because behavioral experiments have shown that invertebrates make decisions that are not always simple and reflexive, but are often the product of careful cost-benefit calculations (Ydenberg and Dill, 1986; Lima and Dill, 1990; Chittka et al., 2009). Thus, invertebrates are ideally suited to study the neural mechanisms underlying value-based decision making. In the following section, we will review some recent experiments on value-based decision making in response to predatory threat, and provide two examples where economic decisions can be linked to identifiable neural circuitry.

### Crayfish

**Figure 6:**
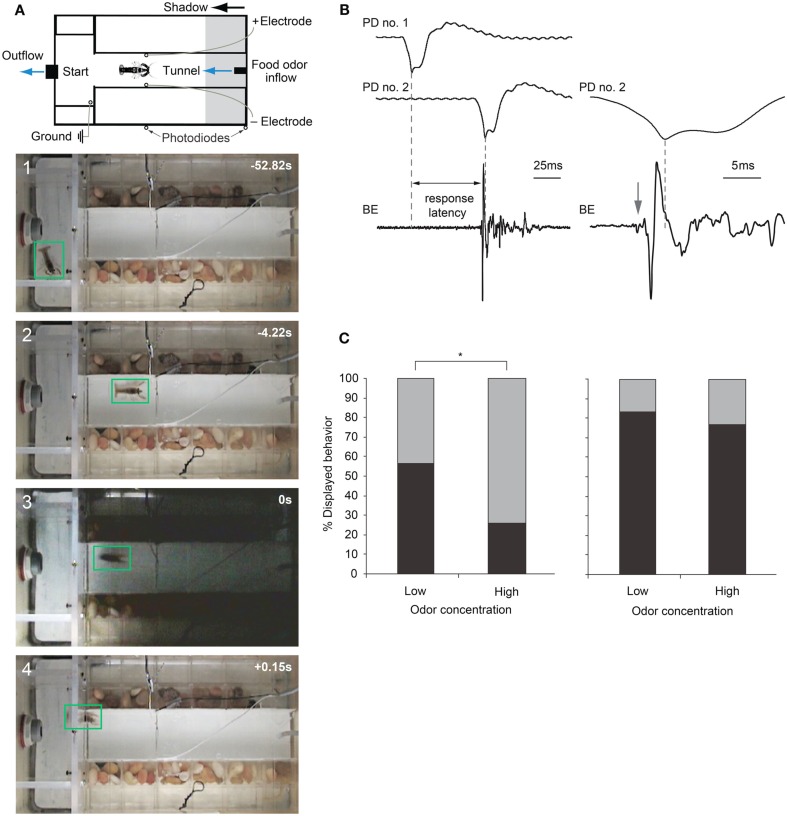
**Escape choices and neural activation in crayfish exposed to approaching shadows**. **(A)** Experimental diagram and four video frames illustrating a crayfish foraging (first two panels) and then tail-flipping (last two panels) in response to a fast approaching shadow with time in seconds. **(B)** Left: example recordings from photodiodes positioned on the tank walls (PD no. 1 and PD no. 2) when a shadow passes by, and from bath electrodes (BE) located inside the tank that capture field potentials generated during a tail-flip. Right: Traces from PD no. 2 and BE at higher temporal resolution. In this example, animal initiated a tail-flip response (arrow) 4 ms before the shadow collided with the animal and produced the peak response in PD no. 2. The first small deflection (arrow) in the BE trace is due to MG neuron activation, while the large phasic potential and the smaller more erratic potentials that follow are due to muscular activity during tail-flips. **(C)** Left: when exposed to a medium speed shadow (2 m/s), crayfish produce fewer tail-flips (black bars) and more freezing (gray bars) when food odor concentration flowing through the tank is high. Right: when exposed to slower (1 m/s) shadows, the effect of food odor concentration on behavioral choice is less pronounced. **(A)** Modified from Liden and Herberholz (2008). **(B,C)** Modified from Liden et al. (2010).

When juvenile crayfish are exposed to fast-moving shadows while foraging in an artificial stream environment, they respond by choosing one of two behavioral actions: they either freeze in place and remain motionless for several seconds before resuming foraging or they produce a tail-flip mediated by the MG neuron that propels the animal backward and away from the approaching shadow and the expected food source (Liden and Herberholz, 2008; Figure 6A). Thus, crayfish respond to visual threat signals that simulate the imminent attack of a predator with defensive behaviors that are discrete and incompatible. When Liden and Herberholz (2008) exposed groups of juvenile crayfish to different shadow velocities, they found that the frequencies of the two behavioral responses were dependent on shadow speed. Slower moving shadows evoked more tail-flips than freezing, but as shadow speed increased the frequency of tail-flips decreased and crayfish primarily produced freezing behavior. The study also showed that different individuals choose different anti-predator strategies when exposed to one type of shadow. Some animals decided to freeze in response to the danger signal while others decided to tail-flip. This suggests that different crayfish have different thresholds for each behavioral action, but what underlies this difference remains to be determined. Because all tested animals were of identical size and shared the same social experiences and feeding history, other intrinsic factors must be responsible.

Recently, Liden et al. (2010) used the same experimental design to show that crayfish base their escape decisions on the values of each behavioral option. They measured escape latencies for shadow-induced MG-mediated tail-flips by comparing photodiode signals with bath electrode recordings that non-invasively captured neural and muscular activity produced during tail-flips (Figure 6B). They found that very fast approaching shadows become inescapable because they collided with the animal before a tail-flip could be generated. Moreover, tail-flips are costly because they move the animal away from the expected food source. Thus, the observed suppression of tail-flipping in favor of freezing in animals facing inescapable shadows, where the value of a tail-flip would be low, reflects the output product of an “economic” decision-making process. Although tail-flipping is considered a less risky strategy when experiencing a predator attack, crayfish also defaulted to freezing behavior when the expected reward became more valuable. When food odor concentration in the artificial stream was increased 10-fold, shadows that evoked mostly tail-flips under standard conditions now generated mainly freezing behavior. Interestingly, if high food value was paired with a strong predator signal (a slow moving shadow) that reliably evoked tail-flips under regular conditions, the behavioral shift toward freezing was less pronounced. Thus, a strong predator signal was able to override the exaggerated food incentive (Figure 6C). This illustrates that crayfish calculate the costs and benefits of different behavioral options and they carefully weigh predation risk against expected reward, eventually selecting the most valuable behavioral choice (Liden et al., 2010). Because these observed tail-flips are always generated by activation of MG neurons and the MG circuit is accessible for neurophysiological and neurochemical experiments, the neural workings underlying value-based decision making in crayfish can now be investigated on the cellular level. This establishes the crayfish as an important new model for studying the neuroeconomics underlying predator avoidance. However, to understand the decision-making process on the network level, identification of interneurons that form the descending visual pathway for freezing behavior will be required.

### Sea slug

The marine snail has been a fruitful model for studying the neural mechanisms underlying decision making and behavioral choice. Using a “competing behaviors” paradigm, early work suggested that different incompatible behaviors were organized in a hierarchical model, each controlled by command-like neurons that produced one behavior while inhibiting others. For example, when the sea slug was feeding, avoidance withdrawal in response to a tactile stimulus was suppressed (Kovac and Davis, 1977). This suppression is caused by identified interneurons that are part of the motor circuit that generates feeding. Thus, feeding behavior takes precedence over withdrawal, while escape swimming dominates most other behaviors, including feeding (Jing and Gillette, 1995). The A1 neurons, a bilateral pair of interneurons located in the cerebropleural ganglion of the snail, are necessary elements of the escape swimming behavior, and their activity also inhibits feeding behavior.

**Figure 7:**
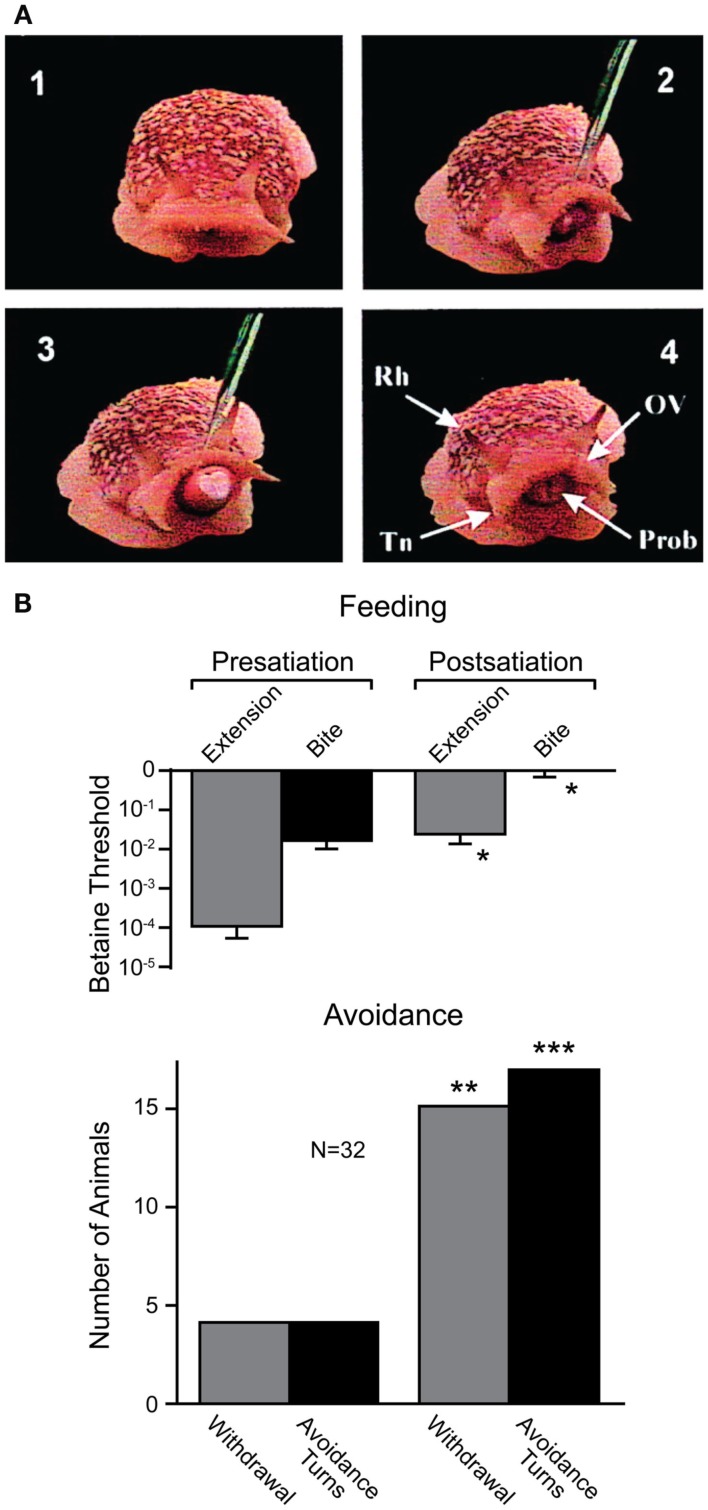
**Effects of internal state on behavioral choice in a sea slug**. **(A)** Four video frames showing feeding behavior in *Pleurobranchaea californica*. Betaine application induces an orienting turn (panel 2) followed by proboscis extension and biting (panel 3). Chemosensory structures (panel 4): rhinophore (Rh), oral veil (OV), tentacle (Tn), and proboscis (Prob). **(B)** Partial satiation raised the threshold for proboscis extension and biting (i.e., feeding), and increased the frequency of withdrawal and turns (i.e., avoidance) in response to betaine. Modified from Gillette et al. (2000).

Recent work, however, has shown that sea slugs base their decisions on cost-benefit computations (Gillette et al., 2000; Figure 7). When presented with food stimuli, feeding behavior or avoidance behavior can be activated, depending on the concentration of the food stimulus and the current behavioral state of the animal. At low concentrations and in satiated animals, food stimuli typically evoked avoidance behavior. When the threshold for feeding was exceeded, avoidance behavior was suppressed, and in hungry snails, even nociceptive stimuli elicited feeding behavior (Figure 7A). This suggests both appetitive and noxious stimuli provide inputs to neural networks underlying feeding and avoidance behavior, but the final behavioral decision is determined by hunger state. Thus, in partially or fully satiated animals, the value placed on feeding behavior is low while it is high for avoidance behavior that protects the animal from predators. Using a simple cost-benefit analysis, the animal weighs nutritional needs against predator risk and selects the most desirable choice (Gillette et al., 2000; Figure 7B). Importantly, feeding and avoidance can be observed as fictive motor patterns in isolated central nervous systems of the snail and some of the neurons controlling these behaviors have been individually identified (Jing and Gillette, 2003). Moreover, in isolated central nervous systems, spontaneous feeding network activity reflects feeding thresholds of the nervous system donors (for proboscis extension and biting); while orienting turns were more frequent in low-feeding threshold donors, avoidance turns dominated in high-feeding threshold donors. When a “command” neuron in the feeding network of a high-feeding threshold donor was electrically stimulated, avoidance turns were converted to orienting turns (Hirayama and Gillette, 2012). Thus, the neurophysiological and neurochemical mechanisms underlying cost-benefit calculations can now be investigated in the isolated nervous system of this animal. This is expected to substantially contribute to our cellular understanding of value-based decision-making processes.

## Conclusion and Future Directions

Recent work in the arthropods discussed suggests that the escape behavior of all may be more complex and varied than has generally been assumed. Quantitative ethograms that divide complex escape maneuvers into a sequence of simpler events can help identify variability within each system. Moreover, combining ethograms with measures of neural structure or neural activity can elucidate the link between discrete motor actions within a series of behavioral events and the corresponding underlying neural mechanisms (Harley et al., 2009; Harley and Ritzmann, 2010).

Based on the high-speed video analysis of the behavior of fruit flies and locusts, a reexamination of the “simple” escape behavior of other arthropods is warranted. Perhaps an analysis at a temporal resolution comparable to that of the speed of production of these behaviors will uncover a degree of flexibility and control not previously appreciated in these animals as well. For example, while the escape tail-flip and freezing behavior of the crayfish in response to visual stimuli have been assumed to be two distinct behaviors, which has been supported by video analysis at 250 fps (Liden et al., 2010), possibly higher speed analysis will show that these distinct decisions are in fact part of a single escape sequence. Such an observation could provide direction in the search for the neural circuit(s) responsible for freezing, the identification of which would provide a unique opportunity to explore decision making between two circuits underlying known behavioral alternatives.

While this new appreciation for the complexity of arthropod escape behavior has reinvigorated work on giant fibers and escape behavior, it raises two significant issues. First, if the giant fiber systems previously assumed to underlie observed escape behaviors are not in fact necessary or sufficient for the production of these behaviors, what circuits are? While Fotowat et al. (2009) have made initial progress toward characterizing the activity of part of an additional putative escape circuit, the neurons will have to be anatomically identified and the circuit fleshed out in future work. Second, if the giant fibers are not involved in escape behaviors produced under existing experimental contexts, what contexts elicit their recruitment? It would be exceedingly wasteful for the largest axons in the fruit fly’s nerve cord to go unused. There must be some combination of internal states and external stimulus conditions that lead to GF-mediated escape response and work should be directed toward identifying these constraints.

It is likely other arthropod models will have a similar redundancy in escape circuitry as has been described in the crayfish. Thus, a comprehensive understanding of decision making during predator avoidance will have to wait until all pathways and not just parts of some are fully characterized (Figure 5). While the identification of all escape circuits in any one arthropod is non-trivial, that parts of both command and non-command systems have been successfully identified in various arthropods is evidence of the feasibility of such a research program. For example, the LG neurons in grapsid crab are fully characterized and individually identifiable cells that can be accessed for intracellular recordings in live animals. The activity of these neurons is highly correlated with behavioral output, which suggests that they play a major role in mediating escape decisions. However, relevant analysis of the complete escape circuit is still missing and descending pathways that orchestrate motor actions need to be identified.

As such, future work should focus on completing the picture of currently known circuits, where often substantial sensory or motor elements remain poorly characterized, as well as identifying unknown but hinted at command or non-command circuits. This hunt for currently uncharacterized circuits might be aided by the possible similarity to and knowledge of already characterized systems found in related species (Figure 5). For instance, the poorly studied non-giant tail-flip circuit in crayfish might share characteristics with that of the DCMD circuit in locusts and knowledge of the structure and function of the DCMD circuit could aid in the identification and characterization of this escape system.

Due to the assumption that giant fiber systems were a singular system responsible for the production of all escape behaviors, there is currently much confusion as to what discrete escape behavior is subserved by what specific circuit. Since it now appears that there are likely many circuits that produce a range of escape behaviors, the spectrum of these behaviors and the stimulus conditions that lead to their display will need to be carefully cataloged and behavioral assays developed that can differentiate them. However, without the ability to simultaneously record both escape behaviors and neural activity, it will be difficult to ascribe a discrete escape sequence or subcomponent of escape behavior to a particular circuit or set of neurons. For this, the use of telemetry that allows for *in vivo* recordings in freely behaving animals (Fotowat et al., 2011; Harrison et al., 2011) will have to be expanded to other invertebrates. While it will be some time before these techniques can be adapted to all models, some should be able to benefit immediately. Arguably, these techniques might have the most to offer in models like the crayfish where large parts of a number of well-described escape circuits have long been worked out (Figure 5A). In such a model, not only can the function of identified neurons be correlated to the performance of distinct components of a complex behavioral sequence, but also how an animal chooses between a range of escape behaviors might be elucidated. Recordings with implanted electrodes or bath electrodes, which non-invasively record neural and muscular field potentials in freely behaving animals, have begun to reveal some of the basic neural patterns underlying escape decisions in crayfish (Herberholz et al., 2001, 2004; Liden and Herberholz, 2008; Liden et al., 2010).

There is a notable lack of neuroethological studies focused on escape mechanisms produced under natural conditions. While staged encounters with natural predators in the laboratory provide some insight into the interplay between neural function and ecologically relevant escape behavior, these studies are sparse. Field studies on the other hand are often focused on ecology and behavior and not designed to investigate neural processes. Occasionally, data sets obtained separately in the field and laboratory allow for a comparative view and for correlating firing patterns of individual neurons and natural escape behavior (e.g., Hemmi and Tomsic, 2012); however, the development of new technologies that permit direct measures of nervous system function in natural settings is highly desirable.

Finally, the neuromodulation of escape behavior by monoamines such as octopamine, serotonin and dopamine is worth further exploration. Although a number of the escape circuits discussed have been shown to be responsive to the application or removal of monoamines (Glanzman and Krasne, 1983; Bustamante and Krasne, 1991; Stern et al., 1995; Pflüger et al., 2004; Harvey et al., 2008; Rind et al., 2008), little is known about the context in which these monoamines affect the performance of behavioral decisions. Since most invertebrate aminergic effects are mediated by metabotropic receptors that can have a gradual but pronounced impact on behavior, monoamines are an attractive candidate for how a nervous system may be biased toward the production of one behavior over another (Crisp and Mesce, 2006; Mesce and Pierce-Shimomura, 2010). Through these monoamines, escape behaviors might modulate or be modulated by competing behaviors. Monoamines (e.g., dopamine and serotonin) have been targeted for roles in decision making and the encoding of punishment and reward (Daw et al., 2002). Thus, the study of monoamines in the context of the evolutionarily critical task of predator avoidance provides an excellent opportunity to explore the postulated neurochemical currency of neuroeconomic decision making. Unfortunately, little work on value-based decision making has been undertaken with invertebrates despite the description of numerous value-based decisions that are likely to involve identified circuits including those mediating escape or avoidance behavior. Research in this field is currently limited to a few invertebrate species, namely the previously discussed sea slug and crayfish, where basic neural mechanisms underlying cost-benefit computations have been partially uncovered. It is surprising that researchers interested in neuroeconomics have not taken greater advantage of these highly tractable models, as they are likely to contribute much to this new field, as they have contributed to neuroscience in general (Clarac and Pearlstein, 2007). Possibly we have just begun to realize that invertebrate models are ideally suited to answer some of the most challenging questions faced today by neuroscience research.

## Conflict of Interest Statement

The authors declare that the research was conducted in the absence of any commercial or financial relationships that could be construed as a potential conflict of interest.
